# New methods drive new biology

**DOI:** 10.1093/genetics/iyaf262

**Published:** 2026-01-07

**Authors:** Konrad Lohse, Stanley Fields, Maria Chikina

**Affiliations:** Institute of Ecology and Evolution, School of Biological Sciences, University of Edinburgh, Edinburgh EH9 3FL, United Kingdom; Department of Genome Sciences, University of Washington, Seattle, WA 98195, United States; Department of Computational and Systems Biology, University of Pittsburgh, Pittsburgh, PA 15213, United States

The many revolutions in high throughput data generation over the last couple of decades have fundamentally transformed genetics: Many, if not most, areas of our field are now largely limited by the availability of methods rather than data. One manifestation of this change is that genetic research is increasingly concerned with datasets that are complete in some sense: e.g. expression variation quantified using single cell atlases or genome-wide variation for entire populations. The methodological limitation of our discipline that results from this is twofold: on the one hand, analysing large volumes of data requires data structures, algorithms and pipelines that are maximally efficient. However, on a more fundamental level, making sense of the new big datasets also requires new concepts, theory and methods. For example, a complete description of population genomic variation is not possible by applying classic population genetic analysis to ever larger datasets. Instead, the challenge of population genomics has spurred a fast-growing field that involves new models and analyses that are framed in terms of haplotypes, tree sequences and ancestral recombination graphs.

GENETICS has a long and fruitful history of publishing seminal methods papers: From Luria and Delbruck's invention of fluctuation analysis to study mutations in bacteria (Luria and Delbrück 1943), invention of the cis-trans test for position effect (Lewis 1945), establishment of new model systems such as *C. elegans* (Brenner 1974), analyses of cell cycle mutants in budding yeast (Hartwell et al. 1973), techniques and assays to measure genetic variation and its effects (Lander and Botstein 1989; Starita et al 2015), to technologies for genome engineering (Gratz et al 2013) and targeted mutagenesis (Christian et al. 2010). On the conceptual side, methodological research in GENETICS includes the fundamental algorithms of modern sample based population genetics (e.g. Tajima 1983; Hudson and Kaplan 1988; Li and Stephens 2003), as well as statistical methods (e.g. Kuhner et al 2000; Meuwissen et al 2001; Nielsen and Wakeley 2001; Beaumont et al 2002; Patterson et al. 2012) and simulation tools (Messer 2013; Baumdicker et al 2022) that connect genetic variation to phenotypic traits and the evolutionary process at the population level.

Our motivation for this first special issue of GENETICS on Methods was to build on this rich canon of work and compile a set of papers in the widest possible sense: a cross-section showcasing current methods advances in all areas of genetics. We hope that the outstanding contributions contained in this special issue will not only help to make better sense of genetic data but also inspire future methods to move genetics research forward.
